# Gamblers seeking treatment: Who does and who doesn’t?

**DOI:** 10.1556/JBA.3.2014.3.7

**Published:** 2014-09-27

**Authors:** BARBARA BRAUN, MONIKA LUDWIG, PAWEL SLECZKA, GERHARD BÜHRINGER, LUDWIG KRAUS

**Affiliations:** ^1^IFT Institut fűr Therapieforschung, Munich, Germany; ^2^Institut fűr Psychologie, Universität Hildesheim, Germany; ^3^Addiction Research Unit, Institut fűr Klinische Psychologie und Psychotherapie, Technische Universität Dresden, Germany; ^4^Centre for Social Research on Alcohol and Drugs (SoRAD), Stockholm University, Sweden

**Keywords:** treatment-seeking, predictors, pathological gamblers, subclinical pathological gamblers, addiction care

## Abstract

*Background and aims:* As only a minority of pathological gamblers (PGr) presents for treatment, further knowledge about help-seeking behavior is required in order to enhance treatment utilization. The present study investigated factors associated with treatment participation in gamblers in Germany. As subclinical pathological gamblers (SPGr, fulfilling one to four DSM-IV-criteria) are target of early intervention due to high risk of transition to pathological gambling, they were subject of special interest. *Methods:* The study analyzed data from a general population survey (n = 234, SPGr: *n* = 198, PGr: *n* = 36) and a treatment study (n = 329, SPGr: *n* = 22, PGr: *n* = 307). A two-step weighting procedure was applied to ensure comparability of samples. Investigated factors included socio-demographic variables, gambling behavior, symptoms of pathological gambling and substance use. *Results:* In PGr, regular employment and non-German nationality were positively associated with being in treatment while gambling on the Internet and gaming machines and fulfilling more DSM-IV-criteria lowered the odds. In SPGr, treatment attendance was negatively associated with married status and alcohol consumption and positively associated with older age, higher stakes, more fulfilled DSM-IV criteria and regular smoking. *Conclusions:* In accordance to expectations more severe gambling problems and higher problem awareness and/or external pressure might facilitate treatment entry. There are groups with lower chances of being in treatment: women, ethnic minorities, and SPGr. We propose target group specific offers, use of Internet-based methods as possible adaptions and/or extensions of treatment offers that could enhance treatment attendance.

## Introduction

Despite pathological gambling (PG) being a disorder with serious adverse consequences in personal, family and professional life (DSM-IV, American Psychiatric Association, 2000), merely up to 10% of pathological gamblers (PGr) seek help (Australian Productivity Commission, 1999; Bischof, Meyer & Bischof, 2012; Cunningham, 2005; Hildebrand, Sonntag, Bauer & Bühringer, 2009; Ladouceur, Lachance & Fournier, 2009; Meyer et al., 2011; National Opinion Research Center, 1999; Suurvali, Hodgins, Toneatto & Cunningham, 2008). The German outpatient addiction care system aims at being easily accessible and also offers early intervention for subclinical pathological gamblers (SPGr; meeting 1-4 DSM-IV criteria, also called diagnostic orphans (Hasin & Paykin, 1998). Despite the offer and the fact that, according to epidemiological studies, SPGr are more prevalent than PGr (Sassen et al., 2011), only about 7% of the outpatient addiction care patients were SPGr (Braun, Ludwig, Kraus, Kroher & Bühringer, 2013).

In sum, the data on help-seeking behavior pose the question whether those presenting for treatment represent a specific subgroup of persons with gambling-related problems. In the field of alcohol dependence research it is suggested that those who seek treatment and those who do not constitute “two worlds of alcohol problems” (Storbjörk & Room, 2008). As there is evidence for etiological and phenomenological similarities between PG and substance-related disorders (Petry, 2006), analogous differences in help-seeking behavior of persons with gambling-related problems are possible. This paper aims at investigating these factors associated with treatment-seeking in this group, providing basis for adapting the addiction care system to increase help-seeking.

There is a growing body of research on motivators for and barriers to seeking help in PGr (Pulford et al., 2009a, 2009b; for an overview see Suurvali, Cordingley, Hodgins & Cunningham, 2009; Suurvali, Hodgins & Cunningham, 2010; Suurvali, Hodgins, Toneatto & Cunningham, 2012a, 2012b); nevertheless, other factors in relation to help-seeking need further investigation. Literature on the subject mostly refers to PGr, leaving a knowledge gap regarding the factors associated with help-seeking in SPGr. Further, growing evidence suggests that SPGr and PGr need to be considered as distinctive groups (Raylu & Oei, 2009). Therefore, the following hypothesized differences between gamblers in treatment and not in treatment were examined for SPGr and PGr separately:

For (1) *socio-demographic characteristics,* it was hypothesized that PGr in treatment are more likely to be male (Volberg, 1994; Weinstock et al., 2011), to have a higher level of education, to belong to the ethnic majority, and to be in a relationship (Volberg, 1994). Because of a latency between symptom development and help-seeking (Toneatto et al., 2008), they are expected to be older than PGr not in treatment.

(2) With regard to *gambling behavior and symptoms of PG,* more severe gambling symptoms were assumed for PGr in treatment (Hodgins & el-Guebaly, 2000; Slutske, Blaszczynski & Martin, 2009; Weinstock et al., 2011), with more DSM-IV criteria fulfilled (Strong & Kahler, 2007), higher stakes across all gambling activities (Nett, Schatzmann, Gerber & Klingemann, 2003), higher gambling frequency, and participation in more gambling activities, as well as in hazardous gambling activities such as slot machines, sports betting and/or online gambling (Meyer, Häfeli, Mörsen & Fiebig, 2010; Sassen et al., 2011).

(3) Rates of *co-morbid substance use disorders* are elevated for PGr in treatment (Nett et al., 2003; Petry, 2005; Petry, Stinson & Grant, 2005; Specker, Carlson, Edmunson, Johnson & Marcotte, 1996). Thus, substance use, i.e. alcohol consumption, illicit drug use, and regular smoking, was assumed to be more present in this group.

## Methods

Two samples of SPGr/PGr were merged and analyzed conjointly.

### General population study (GS)

*Procedure.* Data were taken from the 2006 and 2009 Epidemiological Survey of Substance Abuse (ESA, Kraus & Baumeister, 2008; Kraus & Pabst, 2010), a nationwide cross-sectional household survey conducted in Germany. A representative sample of adults aged 18 to 64 years was drawn from the German general population with a two stage probability sampling design. Younger age groups were over-sampled to account for a lower response probability (disproportional design). After a postal invitation letter informing about the study, a mixed mode design was applied (2006: postal or telephone participation, 2009: online participation was offered additionally). Detailed study description can be found elsewhere (Kraus & Baumeister, 2008; Kraus & Pabst, 2010; Ludwig, Kraus, Müller, Braun & Bühringer, 2012).

*Participants.* A total of *n* = 7,912 and *n* = 8,030 subjects participated in 2006 and 2009, respectively, corresponding to response rates of 44.9% (in 2006) and 50.1% (in 2009) of all eligible subjects. Cases with missing values on lifetime gambling participation (2006: *n* = 81; 2009: *n* = 22) or PG (2006: *n* = 14), inconsistent responses regarding preferred gambling activity (2009: *n* = 2) or implausible values in number of children (e.g. >60 children; 2006: *n* = 6) were excluded. Also, cases of treatment for PG were discarded (2006: *n* = 1; 2009: *n* = 4). The analytical samples comprised *n* = 7,810 (2006) and *n* = 8,002 (2009). The number of persons having participated in any gambling activity within the past year was *n* = 3,582 (2006) and *n* = 3,675 (2009).

### Treatment study (TS)

*Procedure.* The study was carried out in Bavarian outpatient addiction care facilities specialized in counseling/treatment of PG. A convenience sample of patients who presented between April 2009 and August 2010 was drawn consecutively by examining them at the beginning and end of their treatment. Patients gave informed consent to participation in the study. Data collection was twofold: (1)*patient documentation:* facility staff filled in a standardized documentation form for the nationwide statistical report on treatment facilities for substance use disorders in Germany (beginning and end of treatment); (2) *self-report questionnaire,* which patients filled in after the second or third treatment session.

*Participants.* A total of *n* = 466 patients from *n* = 36 facilities was collected. The response rate of the eligible addiction care facilities was 86% (36/42) with an average of *n* = 12 patients participating per facility (*SD* = 11, *R* = 1-45). Based on an extrapolation from an average presentation of *n* = 18 (2009) and *n* =19 (2010) patients per facility (Braun, Kraus, Taqi & Sassen, 2012), the subject participation rate is estimated to be about 50% (466/944). If a patient was willing to participate, but could not fill in the self-report questionnaire, e.g. due to language problems, only data from the patient documentation was collected. This procedure also applied if a patient had given informed consent but presented only once at the facility. Subjects with missing data on age and/or sex (n = 5) were excluded, resulting in an analytical sample of *n* = 461 patients (based on patient documentation). Of these, *n* = 337 patients filled in the self-report questionnaire.

### Measures

In order to assure comparability of the results the same measures were applied in the GS and the TS. Investigated *sociodemographic variables* were sex, age, nationality, marital status, number of children, highest level of education, and employment status.

*Gambling behavior* was assessed as lifetime and 12-month prevalence of participation in all major kinds of gambling available in Germany. Weekly gambling was coded as gambling less than once per week vs. once or several times per week. Multiple gambling was defined as participation in one vs. more than one gambling activity. Stakes were assessed as average stakes in all gambling activities per month within the past year.

*Pathological gambling.* In both studies PG was investigated using a 19-item questionnaire (Stinchfield, 2003) based on the 10 DSM-IV criteria of PG (American Psychiatric Association, 2000). Due to economic reasons, in the GS sample, only participants who had bet at least 50 EUR (about 65 USD) in the past 12 months were asked to answer the questionnaire. Previous findings indicate that individuals betting lower amounts of money are at no or at only a limited risk of fulfilling any DSM-IV criteria for PG (Currie et al., 2006; National Research Council, 1999). Meeting one to four criteria was classified as subclinical pathological gambling (SPG) and five or more criteria as PG.

*Substance use.* Alcohol and tobacco consumption, and illicit drug use (opioids, cannabis, sedatives/hypnotics, cocaine/crack, stimulants/amphetamines/Ecstasy, hallucinogens/LSD, volatile substances, others) within the past 30 days were assessed. Participants were classified as regular smokers when tobacco was consumed on 20 days or more.

### Weighting

In order to achieve representativity, a two-step weighting procedure was applied (see Storbjörk & Room, 2008): (1) *sample weights* were used separately for GS and TS sample to ensure sample representativity for the respective population and (2) a *population weight* was employed in order to fit the conjoint sample of GS and TS to the distribution of SPGr/PGr in treatment vs. SPGr/PGr not in treatment. For the final weight, the two weights were combined multiplicatively (Korn & Graubard, 1999).

*Sample weights.* In the GS, a design weight was computed to account for the disproportional design, i.e. each case was weighted with the reverse of the selection probability (Groves et al., 2009). Additionally, a poststratification weight ensured convergence to the German population aged 18 to 64 years with regard to basic socio-demographic variables (according to the Federal Statistical Office of Germany). The GS sample weight consisted of the multiplied design and poststratification weights. Both for 2006 and 2009, the GS weight’s effectiveness (Korn & Graubard, 1999; Little, Lewitzky, Heeringa, Lepkowski & Kessler, 1997) fall within the typical range of general populations surveys between 60% and 70% (Rösch, 1994), and, therefore, are considered satisfactory. 2006: weight’s range *R* = 0.12-3.49, *SD* = 0.48, effective sample size after weighting *n’* = 6,426 (before weighting *n* = 7,912), effectiveness *E* = 81.2%. 2009: weight ranged from *R* = 0.27-2.99, *SD* = 0.44, effective sample size *n’* = 6,704 (before weighting *n* = 8,030), effectiveness *E* = 83.5%.

In the TS, as no sample was drawn, no design weight was necessary. To adjust socio-demographic characteristics (age, sex, level of education, employment status) of the sample collected in Bavaria (one of 16 German Federal States) to the distribution in the population of treated PGr, a poststratification weight was computed (using iterative proportional fitting/raking; Deming & Stephan, 1940; Gelman & Carlin, 2002). The population consisted of patients treated for PG in all German outpatient addiction care facilities, for which data were available from the statistical report on treatment facilities for substance use disorders in Germany (Steppan, Künzel & Pfeiffer-Gerschel, 2010). The TS weighting was satisfactory [range *R* = 0.24-2.92, *SD* = 0.44, effective sample size of *n’* = 376 (before weighting *n* = 447), effectiveness *E* = 84.1%]. Tables of socio-demographic characteristics of the unweighted and weighted samples and populations for GS and TS are available on request.

*Population weight.* After merging the GS and TS data sets, a population weight (poststratification) was computed in order to map the ratio of SPGr/PGr in treatment and not in treatment. As this study aims at examining SPGr and PGr separately, population weights were computed for both groups. Basis for population weighting was data from a recent general population survey conducted in Germany (PAGE, Meyer et al., 2011). About 10% of PGr enter treatment services; accordingly the weight was computed as 10:90 for PGr in treatment vs. PGr not in treatment. For SPGr, no precise treatment entry rate was reported. Thus, population weighting was estimated based on the following information: (i) the ratio of SPGr vs. PGr having any contact to the addiction care system was 7% vs. 23% and (ii) the ratio of PGr having any contact vs. PGr utilizing treatment more than just marginally was 23% vs. 10%. Assuming that the ratio of having any contact with vs. utilizing treatment is the same for SPGr, the estimation for SPGr utilizing treatment consequently results in 3% (x/7 = 10/23). Thus, a ratio of 3:97 for SPGr in treatment vs. SPGr not in treatment was used for weighting.

*Combined weight.* The multiplied sample and population weight for SPGr was satisfactory (R = 0.18-2.27, *SD* = 0.53, effective sample size *n’* = 172, E = 78%). The combined weight for PGr had a range of *R* = 0.03-13.17 and a standard deviation of *SD* = 2.59. The effective sample size was *n’* = 38, the effectiveness *E* = 11.1%, which is unsatisfactory. However, the weight was not trimmed (e.g. at the 95th percentile) in favor of a more realistic mapping of the ratio of PGr in treatment vs. PGr not in treatment. The problem was handled by employing sensitivity analyses (see below).

### Statistical analyses

Stata/SE 12.0 (StataCorp LP, 2007) was used for all analyses and analyses were conducted separately for SPGr and PGr.

The *dependent variable* treatment status was operationalized by samples “in treatment” (TS-sample) and “not in treatment” (GS-sample; subjects in treatment within the past 12 months had been excluded from the analyses, *n* = 5).

*Descriptive analyses* were conducted for the samples in treatment vs. not in treatment and are presented in Table 1. For each of the included variables, differences in distribution were estimated using Pearson’s x^2^-tests or exact Fisher-test (categorical variables) and *t*-tests (interval scaled variables).

*Logistic regressions* were employed to identify predictors of treatment status among socio-demographic variables, gambling behavior, number of fulfilled PG diagnostic criteria, and substance use. Gambling participation in hazardous gambling activities (any Internet gambling, casino games, gaming machines) and participation in lotteries were included. The latter was introduced as it is the most common gambling activity in Germany (Bundeszentrale für gesundheitliche Aufklärung, 2012; Meyer et al., 2011; Sassen et al., 2011). Collinearity was analyzed and no predictor was excluded (variance inflating factor (VIF) <10, tolerance >0.2). Blockwise hierarchical regression with backward elimination was used: (1) substance use, (2) gambling behavior and PG, and (3) socio-demographic variables were tested with a Wald x^2^-test in the overall model and removed from the model if failing to contribute to variance explanation. Goodness of fit was tested with the *F-adjusted mean residual test* (Archer, Lemeshow & Hosmer, 2007) suitable for weighted data.

*Sensitivity analyses* for PGr were conducted because of non-effective final weights, resulting in three different models employing (1) the final weight (multiplied sample and population weight), (2) the sample weight (good effectiveness) , and (3) without any weight. Due to Bonferroni adjustment, the α-level was set at .017 for the overall models. Only stable predictors and blocks of predictors, i.e. significant in all models, were considered robust.

### Ethics

Both the TS and the GS were approved by the Ethical Board of the German Society of Psychology (DGPs; GS: Reg.-No: GBLK06102008DGPS; TS: Reg.-No: LK 12.2008). All participants were informed about the study and provided informed consent.

## results

A total of *n =* 329 were in treatment (SPGr: *n =* 22; PGr: *n =* 307) and *n =* 234 were not in treatment (SPGr: *n =* 198; PGr: *n =* 36).

### Subclinical pathological gamblers

*Descriptive analyses* revealed differences between SPGr in treatment (n = 22) und SPGr not in treatment (n = 198; Table 1) with SPGr in treatment having higher level of education and higher unemployment rate, higher stakes in the past year and higher number of fulfilled DSM-IV criteria for PG. Being in treatment was associated with participation in lotteries to a lower extent and participation in gaming machines to a greater extent, as well as with alcohol consumption within the past 30 days to a lower extent.

**Table 1.** Descriptive characteristics of SPGr in treatment and SPGr not in treatment

Socio-demographic variables	Not in treatment *n* = 198, %	In treatment *n* = 22, %	p*
Age *[M(SE)]*		40(1.0)	42 (3.0)	.504
Sex	male	81.3	2.6	.563
	female	15.8	0.3	
Marital status	single	38.8	1.2	.054
	married	50.1	0.9	
	divorced/widow	ed 8.3	0.7	
Education level^1^	low	39.0	1.4	**.037**
	medium	1.3	26.1	
	high	0.2	33.5	
Employment status	unemployed	2.4	0.4	**.018**
	employed	94.7	2.5	
Nationality	German	79.2	2.7	.216
	other	18.0	0.2	
Gambling behaviour/PG^2^				
Weekly^3^	no	38.2	1.0	.721
	yes	58.9	1.9	
Multiple^4^	no	31.6	1.0	.824
	yes	65.7	1.8	
Number of games [M *(SE)]*	2.4 (0.1)	2.5 (0.4)	.766	
Internet gambling^5^	no	71.2	1.8	.328
	yes	25.9	1.1	
Casino^6^	no	76.1	2.0	.323
	yes	21.1	0.9	
Gaming machines	no	78.6	1.4	**<.001**
	yes	18.2	1.8	
Lotteries	no	17.9	1.5	**.001**
	yes	79.2	1.4	
All hazardous games^7^	no	95.0	2.6	.097
	yes	2.1	0.2	
Stakes [*M* (SE)]^8^		216(81.4)	1,544(509.3)	**.011**
Number of DSM-IV criteria *[M (SE)]*	1.7 (0.1)	3.3 (0.2)	**<.001**
Substance use^9^				
Alcohol	no	25.6	1.4	**.032**
	yes	71.5	1.4	
Smoking^10^	no	5.9	0.1	.595
	yes	91.2	2.8	
Illicit drugs^11^	no	91.9	2.8	.520
	yes	5.2	0.1	

*Notes:* SPGr = subclinical pathological gamblers; for each variable contingency table for SPGr in treatment and not in treatment; *M* = mean; *SE* = standard error; * p-value of x^2^-, Fisher- or t-test*, p* ≤ .05: bold; ^1^low: 9 years of school education/without graduation/other; me- dium: 10 years of school education; high: more than 10 years of school education; ^2^in the past year; ^3^gambling once or several times per week; ^4^participation in one or more gambling activities; ^5^Internet casino, Internet card games, Internet sports betting; ^6^slot machines or table games; ^7^participating in any hazardous game (Internet games, casino, gaming machines); ^8^average stakes per month in the past year for all gambling activities; ^9^in the past 30 days; ^10^regular smoking (≥20 days); ^11^any illicit drug.

All predictors were used for the *blockwise hierarchical logistic regression* with the criterion variable treatment status and all blocks contributed to variance explanation (Table 2). Being in treatment, was predicted by higher age, higher stakes, a higher number of fulfilled DSM-IV criteria and regular smoking (all OR > 1). The odds for being in treatment were lower when marital status was “married” as opposed to “single” and in case alcohol had been consumed within the past 30 days (OR < 1). Although the goodness of fit test was significant (indicating no good model fit), the overall model was significant with a Pseudo *R*^2^ of 67.6.

### Pathological gamblers

*Descriptive analyses.* A higher proportion of PGr in treatment (*n* = 307) were unemployed, gambled at gaming machines, set higher stakes in the past year and fulfilled more DMS-IV criteria for PG compared to PGr not in treatment (n = 36), whereas being in treatment was associated with alcohol consumption within the past 30 days to a lower extent (Table 3).

All predictors were usable for *blockwise hierarchical logistic regression* on treatment status (VIF <10, tolerance > 0.2). Because of low cell populations of female sex and marital status “divorced/widowed” (for both: *n* = 1) in PGr not in treatment, these predictors were not included.

After *sensitivity analyses* the substance use block was included in only two models (Table 4). Treatment status was predicted robustly by socio-demographic variables and gambling behavior and PG. Odds for being in treatment were lower for regular employment and non-German nationality. PGr participating in any Internet game and using gaming machines and those with more fulfilled DSM-IV-criteria had higher odds for being in treatment. Though goodness of fit tests did not indicate good fit, all models reached significance with Pseudo *R*^2^ ranging from 39.5 to 58.5.

## Discussion

As expected, differences between SPGr/PGr in treatment and not in treatment were found. Likewise, SPGr and PGr were found to differ with regard to predictors for treatment status. For SPGr, married persons had a smaller chance of being in treatment compared to singles; older persons had better chances. Also, the chance of being in treatment was higher if stakes gambled were higher, a greater number of DSM-IV criteria were met, or the person smoked regularly, whereas alcohol consumption reduced the odds. In PGr, sensitivity analyses revealed that socio-demographic variables and factors related to gambling behavior and PG were stable predictors of treatment status, whereas substance use cannot be considered a stable predictor. PGr with regular employment and non-German nationality had lower odds for being in treatment, while higher odds were found for PGr gambling on the Internet and gaming machines and for PGr with more symptoms of PG (DSM-IV-criteria). Predictors of treatment status in SPGr/PGr refer to (1) *socio-demographic characteristics,* where the expected sex differences in treatment status were not observed. This may be due to the small size of the female PGr sample (n = 33), especially as a tendency conform to the hypothesis was found in the SPGr sample (females *n* = 40). Contrary to the expectation that help-seeking would be more probable given a stable socio-demographic background, odds for being in treatment were lower for married persons (SPGr) and for persons with regular employment (PGr). A possible explanation is, that gamblers who do not experience far-reaching adverse consequences, e.g. losing their spouse or job, do not seek help solely for gambling-related problems. This is in line with previous findings suggesting that gamblers seek treatment in situation of crisis (Evans & Delfabbro, 2005). Also, having regular employment may be difficult to reconcile with high intensity treatment.

**Table 2.** Blockwise hierarchical logistic regression on treatment status of SPGr

*n* =183						
Predictor	*OR* (95% CI)	*SE*	*z*	p*	p**
Socio-demographic						**.001**
Age	1.18	(1.04-1.34)	0.08	2.51	**.013**	
Sex (ref. male)	0.00	(0.00-1.57)	0.00	-1.85	.066	
Marital status (ref. single)						
married	0.06	(0.00-0.91)	0.08	-2.04	**.043**	
divorced/widowed	2.39	(0.08-68.17)	4.06	0.51	.608	
Education level (ref. low)^1^						
medium	3.38	(0.44-26.06)	3.50	1.17	.242	
high	0.17	(0.00-92.89)	0.53	-0.56	.577	
Employment status (ref. unemployed)	0.51	(0.02-14.91)	0.87	-0.40	.693	
Nationality (ref. German)	0.15	(0.00-4.84)	0.26	-1.08	.282	
Gambling behaviour/PG^2^						**<.001**
Weekly^3^	2.07	(0.10-43.15)	3.19	0.47	.636	
Multiple^4^	1.16	(0.06-23.28)	1.76	0.10	.922	
Number of games	1.22	(0.46-3.21)	0.60	0.41	.684	
Internet gambling^5^	3.46	(0.22-53.53)	4.80	0.89	.373	
Casino^6^	0.77	(0.11-5.24)	0.75	-0.27	.784	
Gaming machines	5.78	(0.37-89.95)	8.05	1.26	.209	
Lotteries	0.13	(0.01-1.88)	0.18	-1.51	.133	
All hazardous games^7^	0.66	(0.01-47.91)	1.43	-0.19	.847	
Stakes^8^	1.00	(1.00-1.00)	0.00	3.33	**.001**	
Number of DSM-IV criteria	4.06	(1.23-13.43)	2.46	2.31	**.022**	
Substance use^9^						**.003**
Alcohol	0.01	(0.00-0.21)	0.01	-2.84	**.005**	
Smoking^10^	37,823	(91-15.7*10^6^)	11,5515	3.45	**.001**	
Illicit drugs^11^	0.09	(0.00-611.96)	0.40	-0.54	.590	
*C* (21)^12^	62.6					
p^13^	**<.001**					
Pseudo R^2^	67.6					
F(9,174)^14^	127.1					
p^15^	**<.001**					

*Notes:* SPGr = subclinical pathological gamblers; *OR* = Odds Ratio; 95% CI = 95% confidence interval; *SE* = robust standard error; * p-value of pre- dictor, ** p-value of block of predictors (Wald x^2^-test); *p <* = .05: bold; ^1^low: 9 years of school education/without graduation/other; medium: 10 years of school education; high: more than 10 years of school education; ^2^in the past year; ^3^gambling once or several times per week; ^4^participation in one or more gambling activities; ^5^Internet casino, Internet card games, Internet sports betting; ^6^slot machines or table games; ^7^participating in any hazardous game (Internet games, casino, gaming machines); ^8^average stakes per month in the past year for all gambling activities; ^9^in the past 30 days; ^10^regular smoking (<20 days); ^11^any illicit drug; ^12^ x^2^-test of model, in braces: degrees of freedom df; ^13^p-value of goodness of x^2^-test; ^14^*F*-value of goodness of fit test, in braces: df; ^15^p-value of goodness of fit test.

As expected, the chance for being in treatment was higher for older persons (SPGr) and lower for persons with non-German nationality (PGr). Older age may reflect a longer problem duration which may be associated with increased problem awareness and greater external pressure to seek help. Further on, language difficulties and cultural barriers could hamper treatment entry and utilization among persons with a non-German nationality (Baschin, Ülsmann, Jacobi & Fydrich, 2012).

(2) In line with expectations for *gambling behavior and PG,* SPGr/PGr in treatment indicated more gambling-related problems. Although gambling frequency and multiple gambling were not associated with treatment status, the chance for being in treatment was higher if more DSM-IV- criteria were fulfilled (SPGr and PGr), treatment odds rose with higher stakes (SPGr) and with participation in Internet gambling and gaming machines (PGr). One could assume that providers of these games carefully monitor persons with possible gambling problems and advise treatment. Then again this can be ruled out as not one patient of the TS had been referred to treatment by a gambling provider (Braun et al., 2013).

(3) Regarding higher *substance use* of PGr in treatment the assumed global connection between substance use and being in treatment was not found. In the SPGr sample, regular smoking had the expected effect (predicting being in treatment). Contrary results were found for alcohol use: about half of the SPGr in treatment were alcohol abstinent; this group had higher odds for being in treatment than consumers. Given the high co-morbidity of alcohol use disorders and PG (Petry, 2006) and assuming that this link also applies for SPG, a possible explanation could be that some SPGr had had alcohol related disorders and reduced their alcohol consumption beforehand. This could have resulted in higher addiction problem awareness and/or higher self-efficacy to be able to solve gambling-related problems, thus leading to a higher willingness to seek help. This interpretation, however, cannot be tested with the available data, especially as (former) diagnoses of alcohol use disorders were not assessed in the TS and could therefore not be used as a predictor.

**Table 3.** Descriptive characteristics of PGr in treatment and PGr not in treatment

	Not in treatment	In treatment	p*
Socio-demographic variables		*n* = 36, %	*n* = 307, %	
Age *[M(SE)]*		33 (2.2)	36 (0.7)	.096
Sex	male	86.4	10.0	.159
	female	2.5	1.1	
Marital status	single	53.8	6.2	.064
	married	33.0	3.1	
	divorced/widowed	2.4	1.6	
Education level^1^	low	28.3	7.1	.088
	medium	47.6	4.4	
	high	10.7	2.0	
Employment status	unemployed	2.0	3.6	**<.001**
	employed	86.5	8.0	
Nationality	German	65.1	9.4	.120
	other	23.8	1.7	
Gambling behaviour/PG^2^				
Weekly^3^	no	19.8	1.6	.266
	yes	69.2	9.4	
Multiple^4^	no	25.0	2.3	.426
	yes	64.1	8.5	
Number of games [M *(SE)]*		2.9 (0.3)	2.8 (0.1)	.881
Internet gambling^5^	no	71.0	8.3	.487
	yes	17.9	2.8	
Casino^6^	no	57.2	7.0	.863
	yes	31.7	4.1	
Gaming machines	no	31.7	1.9	**.013**
	yes	56.6	9.9	
Lotteries	no	39.3	4.5	.723
	yes	49.6	6.6	
All hazardous games^7^	no	80.5	10.1	.907
	yes	8.4	1.0	
Stakes [M(SE)]^8^		444(123)	2,352 (316)	**<.001**
Number of DSM-IV criteria *[M (SE)]*	6.7 (0.3)	7.9 (0.1)	**<.001**
Substance use^9^				
Alcohol	no	22.3	6.1	**.002**
	yes	66.6	5.0	
Smoking^10^	no	3.5	0.3	.616
	yes	85.4	10.8	
Illicit drugs^11^	no	79.0	10.5	.709
	yes	9.5	1.0	

*Notes:* PGr = pathological gamblers; for each variable contingency table for PGr in treatment and not in treatment, no *n* are reported but proportions after weighting; M = mean; SE = standard error; * *p*-value of *x*^2^-, Fisher- or *t*-test, *p*≤ .05: bold; ^1^low: 9 years of school education/without gradua- tion/other; medium: 10 years of school education; high: more than 10 years of school education; ^2^in the past year; ^3^gambling once or several times per week; ^4^participation in one or more gambling activities; ^5^Internet casino, Internet card games, Internet sports betting; ^6^slot machines or table games; participating in any hazardous game (Internet games, casino, gaming machines); ^8^average stakes per month in the past year for all gambling activi- ties; ^9^in the past 30 days; ^10^regular smoking (≥20 days); ^11^any illicit drug.

### Limitations

Though being potentially important predictors of help-seeking several variables, e.g. comorbid psychiatric disorders, personality traits or duration of PG, were not assessed in order to keep study participation effort as low as possible.

Other limitations could have compromised representativity and generalizability of the results. The response rates of about 50% in the GS sample may have resulted in a selection bias. Also, the weighted sample still differed from the general population with regard to nationality and level of education (in 2006 and 2009). Furthermore, a mode effect for the different questionnaire modes (paper, telephone, online) was found. These limitations and others are discussed in Kraus and Baumeister (2008) and Kraus and Pabst (2010).

Generalizability of the TS may be compromised due to a response rate of 50% and data having been obtained from only one of the 16 Federal States of Germany. However, socio-demographic and treatment characteristics of the TS resemble those of PGr treated in all of Germany (based on the statistical report of treatment facilities in Germany) and socio-demographic characteristics were additionally accounted for by weighting. Thus, a selection bias is unlikely. Also, no selection bias due to non-response (17%) of eligible addiction care facilities needs to be assumed. Still, not having included all Bavarian addiction care facilities as eligible may have compromised the general representativity of the sample. This was for pragmatic reasons, as case numbers in facilities not specialized in counseling/treatment of PG were expected to be low.

**Table 4.** Blockwise hierarchical logistic regression and sensitivity analyses on treatment status of PGr

*n* = 258				Model with final weight														Model with sample weight													Model without any weight								
Predictor			*OR* (95% Cl)			*SE*			*z*			p*			p** OR (95% Cl)								*SE*	*z*	p*				p** OR (95% Cl)					*SE*			*z*	p*	p**
Socio-demographic														**<001**														**<001**									**.004**		
Age			1.15	(1.05-1.25)	0.05			3.06			.002			1.05				1.00-1.11				0.03	1.89	.058				1.08	0.99-1.17			0.05			1.80	.072			
Sex (ref. male)			-	-	-			-			-			-				-				-	-	-				-	-			-			-				
Marital status (ref. single)																																							
married			1.07	(0.14-8.02)	1.09			0.07			.948			0.53				0.14-2.05				0.36	-0.93	.354				0.57	0.14-2.36			0.41			-0.77	.441			
divorced/widowed			-	-	-			-			-			-				-				-	-	-				-	-			-			-				
Education level^1^ (ref. low)																																							
medium			0.11	(0.02-0.54)	0.09			-2.75			**.006**			0.42				0.13-1.37				0.25	-1.44	.150				0.27	0.08-0.95			0.17			-2.04	**.042**			
high			0.03	(0.00-0.48)	0.04			-2.5			**.013**			0.53				0.11-2.48				0.42	-0.81	.416				0.38	0.06-2.25			0.35			-1.06	.287			
Employment status			0.00	(0.00-0.01)	0.00			-4.76			**<.001**			0.06				0.01-0.26				0.04	-3.69	**<.001**				0.10	0.02-0.45			0.08			-2.99	**.003**			
(ref. unemployed)																																							
Nationality (ref. German)			0.06	(0.01-0.49)	0.06			-2.62			**.009**			0.20				0.06-0.66				0.12	-2.66	**.008**				0.17	0.05-0.62			0.11			-2.68	**.007**			
Gambling behaviour/PG^2^														**<.001**														**.012**									**.001**		
Weekly^3^			0.10	(0.02-0.58)			0.09			-2.59			**.010**				0.52			0.12-2.37	0.40				-0.84			.400	0.51				0.10-2.55	0.42				-0.82	.414	
Multiple^4^	0.91	(0.07-12.52)			1.21			-0.07			.945				0.72			0.09-5.39	0.74				-0.33			.745	1.25				0.19-8.19	1.20				0.23	.817			
Number of games	0.76	(0.30-1.97)			0.37			-0.56			.575				0.54			0.29-1.03	0.18				-1.86			.063	0.51				0.26-1.01	0.18				-1.94	.052			
Internet games^5^	552.98	(16-18.412)			984.4			3.55			**<.001**				7.60			1.17^9.39	7.26				2.12			**.034**	13.55				2.06-89.18	13.03				2.71	**.007**			
Casino^6^	5.55	(0.56-54.72)			6.45			1.47			.142				2.16			0.24-19.39	2.42				0.69			.493	1.50				0.24-9.52	1.42				0.43	.664			
Gaming machines	46.02	(2.48-854)			68.25			2.58			**.010**				6.22			1.32-29.24	4.91				2.31			.021	7.31				1.37-39.01	6.25				2.33	**.020**			
Lotteries	1.12	(0.16-8.05)			1.12			0.11			.909				4.97			0.45-54.40	6.07				1.31			.189	5.46				0.73-4.0.62	5.59				1.66	.097			
All hazardous games^7^	0.00	(0.00-0.16)			0.01			-2.84			**.005**				0.19			0.01-3.59	0.29				-1.10			.270	0.17				0.01-4.24	0.28				-1.08	.282			
Stakes^8^	1.00	(1.00-1.00)			0.00			2.83			**.005**				1.00			1.00-1.00	0.00				1.28			.199	1.00				1.00-1.00	0.00				1.24	.215			
Number of DSM-IV	4.12	(2.33-7.29)			1.19			4.89			**<.001**				1.69			1.06-2.71	0.41				2.19			**.028**	1.81				1.11-2.96	0.45				2.36	**.018**			
criteria																																								
Substance use^9^															**.001**												**.126**													**.032**
Alcohol	0.04				(0.01-0.21)			0.03			-3.9			**<.001**	-	-			-			-				-				0.25	0.07-0.86			0.16	-2.19	**.029**			
Smoking^10^	1.06				(0.03-36.05)			1.90			0.03			.973	-	-			-			-				-				1.70	0.16-17.71			2.03	0.45	.656			
Illicit drugs^11^	0.30				(0.03-2.55)			0.32			-1.11			.266	-	-			-			-				-				0.38	0.08-1.80			0.30	-1.22	.224			
											Z^2^(19)12				57.7							Z^2^ (16)12								50.6					X^2^(19)^12^	51.8			
											*P*				**<.001**							*P*								**<.001**					*P*	**<.001**			
											Pseudo *R^2^*				58.5							Pseudo *Ŕ^2^*								39.5					Pseudo R^*2*^	44.7			
											*F(9.*249)^13^				399.5							*F(9.*250)^13^								164.3					F(9.249)13	67.6			
											14 *P*				**<.001**							14 *P*								**<.001**					14 *P*	**<.001**			

*Notes:* PGr = pathological gamblers; *OR =* Odds Ratio; 95% Cl = 95% confidence interval; *SE =* robust standard error; * p-value of predictor, ** p-value of block of predictors (Wald x^2^-test); *p* ≤.05: bold; ^1^low: 9 years of school education/without graduation/other; medium: 10 years of school education; high: more than 10 years of school education; ^2^in the past year; ^3^gambling once or several times per week; ^4^rticipation in one or more gambling activities; ^5^internet casino, Internet card games, Internet sports betting; ^6^slot machines or table games; participating in any hazardous game (Internet games, casino, gam- . ing machines); ^8^average stakes per month in the past year for all gambling activities; in the past 30 days; ^10^regular smoking ( ≥20 days); ^11^any illicit drug; ^12 ^x^2^-test of model, in braces: degrees of freedom df; ^13 ^*p-*value of goodness of x^2^-test; ^14^*F-*value of goodness of fit test, in braces: df; ^15^ p-value of goodness of fit test.

Procedure of the TS included administration of the self-report questionnaire after the second or third treatment session. Up to this point, some study participants had already dropped out of treatment, thus not providing self-reported information. However, a comparison of socio-demographic characteristics did not show any differences.

Another potential limitation results from the weighting procedure. The population weight for the PGr resulted in a low effective sample size. Sensitivity analyses were conducted to account for this problem, and only results with sufficient stability were included in the interpretation.

## Conclusions

The aim of this study was twofold: first, to identify factors associated with help-seeking behavior both in SPGr and PGr, and second, to provide a theoretical basis for possible adaptations of the addiction care system. The extension of treatment offers may contribute to enhancing treatment-seeking and treatment utilization among persons with gambling-related problems. This may be especially necessary for those who only present for treatment to a low extent: Women, persons of an ethnic minority, and SPGr.

*Target group specific offers* focus on the first two groups. For women, gambling-related treatment services should be community-based and available in general health care centers, where seeking help and counseling/treatment might be discreet (Crisp et al., 2004). With regards to the therapy setting, group therapy offers are recommended, taking into account particularities in interpersonal relationships and communication of female PGr (Vogelgesang, 2004). These groups should be available as “women-only” groups to provide a safe space for disclosure (Piquette-Tomei, Norman, Dwyer & McCaslin, 2008). For PGr belonging to an ethnic minority, therapists specialized in cultural sensitive therapy (Raylu & Oei, 2004) or having the same ethnic background reduce the risk of language or cultural barriers hindering treatment-seeking and -utilization. Awareness of treatment options in this group could be enhanced by providing information material and telephone or online offers in different languages, as well as advertising these offers e.g. in religious communities or special services for migration.

*Early identification and early intervention* are crucial to prevent SPGr from possible progressing to PG and experiencing related adverse consequences. One early intervention approach is to offer more specific prevention programs within the existing addiction care systems. But despite already being a target group, SPGr only present to a low extent in addiction care. Hence, new approaches to get access to SPGr are required. The Internet seems a promising possibility, especially as participation in online gambling is increasing (Ludwig et al., 2012). Addressing gamblers in their “natural environment” could facilitate their readiness to participate in early intervention measures. And in light of the general trend to gather health care information online (Baker, Wagner, Singer & Bundorf, 2003), the Internet might help to raise problem awareness.

In line with this, more *information and education* may be a central factor to bring more persons with gambling-related problems into treatment, as problem awareness and knowledge of treatment options are necessary preconditions for help-seeking (Suurvali et al., 2012b). In Germany, for example, knowledge on treatment options is quite low: in 2011, less than 20% of the general population indicated familiarity with an addiction care facility that offers gambling-specific treatment, and less than 10% knew of a gambling helpline telephone number [Bundeszentrale für gesundheitliche Aufklärung (BZgA), 2012]. Nevertheless, compared to 2007, a trend of rising problem awareness and knowledge of treatment offers is obvious (BZgA, 2012). This may be due to increased efforts in the public relations work of public health organizations associated with PG, e.g., engaging in media campaigns and informing about treatment options and telephone helplines (the latter are obligatory on lottery tickets and advertisements since 2008).

Being mainly structural, these measures aim at bringing more persons with gambling-related problems into treatment. In addition to adapting treatment offers, the treatment process itself should be tailored according to the clientele’s needs. In light of treatment drop-out rates ranging from 14% to 50% (Melville, Casey & Kavanagh, 2007), more research on factors influencing treatment utilization is required. Understanding factors influencing not only entering but also staying in treatment is crucial for adapting the addiction care system to better help persons with gambling-related problems.
