# Erratum

**DOI:** 10.1556/JBA.3.2014.4.9

**Published:** 2014-12-18

To the paper:

## Gamblers seeking treatment: Who does and who doesn’t?

BARBARA BRAUN, MONIKA LUDWIG, PAWEL SLECZKA, GERHARD BÜHRINGER and LUDWIG KRAUS

published in the *Journal of Behavioral Addictions* 3(3), pp. 189–198 (2014), DOI: 10.1556/JBA.3.2014.3.7

On page 196 by mistake information regarding the *Conflict of interest* was provided in the *Funding sources* section. The correct statements for the *Funding sources* and the *Conflict of interest* sections are as follows:

*Funding sources:* The treatment study has been funded by the Bavarian State Ministry of Finance, Regional Development and Regional Identity via the Bavarian State Ministry of Public Health and Care Services in the context of the “Bavarian Coordination Center for Gambling Issues” (LSG Bayern). Funding of the general population study (Epidemiological Survey of Substance Abuse: ESA) was provided by the German Federal Ministry of Health (Grant No. 119-4914-8/32). In both cases funding is not subject to any conditions.

*Conflict of interest:* Barbara Braun, Monika Ludwig and Pawel Sleczka have no conflicts of interest to declare. Research by Ludwig Kraus and Gerhard Bühringer has been funded by the Bavarian State Ministry of Finance, Regional Development and Regional Identity via the Bavarian State Ministry of Public Health and Care Services. The Bavarian State Ministry of Finance, Regional Development and Regional Identity serves as regulatory authority for public and private gambling services and as operator of the State Gambling Monopoly. Research by Gerhard Bühringer has also been funded by the commercial gaming industry. No funding was subject to any conditions.

